# Radiological Evaluation of Cervical Spine Involvement in Rheumatoid Arthritis: A Cross-Sectional Retrospective Study

**DOI:** 10.3390/jcm10194587

**Published:** 2021-10-05

**Authors:** Mateusz Kotecki, Robert Gasik, Piotr Głuszko, Iwona Sudoł-Szopińska

**Affiliations:** 1 Department of Radiology, National Institute of Geriatrics, Rheumatology and Rehabilitation, 02-637 Warsaw, Poland; sudolszopinska@gmail.com; 2 Department of Neuroorthopaedics and Neurology, National Institute of Geriatrics, Rheumatology and Rehabilitation, 02-637 Warsaw, Poland; robert.gasik@spartanska.pl; 3 Department of Rheumatology, National Institute of Geriatrics, Rheumatology and Rehabilitation, 02-637 Warsaw, Poland; zruj@mp.pl

**Keywords:** cervical spine, rheumatoid arthritis, atlanto-axial subluxation, radiography, magnetic resonance imaging

## Abstract

Background: Cervical spine lesions are a common manifestation of rheumatoid arthritis (RA). The purpose of this study was to conduct a retrospective analysis of radiological lesions in cervical spine in patients with RA and to correlate findings with clinical and laboratory parameters. Methods: Overall, 240 consecutive patients with RA were referred for imaging by clinicians based on symptoms suggesting cervical spine involvement and/or long disease duration. In each patient, lateral radiographs and MRI of the cervical spine were performed. The imaging data were correlated with clinical records and laboratory data. Results: The cervical spine was affected in 179 patients (75%). The most common lesions were anterior atlanto-axial subluxation (AAS; 58%), subaxial subluxation (58%), and demineralization (48%). Cervical spine involvement was linked to longer disease duration (*p* = 0.007), the presence of rheumatoid factor (RF; *p* = 0.010), elevated C-reactive protein (CRP) levels (*p* = 0.016), and accelerated erythrocyte sedimentation rate (ESR; *p* = 0.025). Longer disease duration was associated with anterior AAS (*p* = 0.005), subaxial subluxation (*p* = 0.005), and basilar settling (*p* = 0.003). Conclusions: As many as 75% of RA patients develop lesions that can be observed on radiographs and through MRI. The most frequent radiological findings include anterior AAS and subaxial subluxation. Long disease duration, RF seropositivity, and elevated inflammatory markers were risk factors for cervical spine involvement.

## 1. Introduction

Rheumatoid arthritis (RA) is an immune-mediated inflammatory disease affecting numerous joints of the peripheral and axial skeleton. Cervical spine involvement is the third most common manifestation of RA after hands and feet and may develop in 17–88% of patients [1]. Atlanto-axial level is the most frequent cervical spine location of RA with possible life-threating complications. Chronic inflammation at the C1/C2 joint may lead to the progressive destruction of bones and ligaments causing subluxations [1], with further cervical spine compression and even sudden death [2]. At the subaxial level, the most prevalent lesions are subluxations, but bony ankylosis or spinous process erosions may also occur [1]. The main risk factors for cervical spine involvement in RA include early onset of RA, advanced disease in peripheral joints, presence of rheumatoid factor (RF), and chronic use of corticosteroids [3,4].

Imaging plays an important role in the diagnosis of cervical spine pathologies, with classic radiography being a first-line approach. Magnetic resonance imaging (MRI) and computer tomography (CT) are used for more precise evaluation of bones and soft tissues. MRI has the possibility to show early inflammatory changes such as effusions, synovitis, bone marrow edema (BME), and the relation of spinal lesions to brain stem, spinal cord, and nerve roots, whereas CT is the most precise technique for complex bone anatomy.

In the last two decades, biological agents have been introduced, and awareness of early RA diagnosis and treatment to prevent chronic complications has grown. Some studies suggest that early aggressive treatment with disease-modifying antirheumatic drugs (DMARDs) may prevent the development of new lesions but does not prevent the progression of existing ones [5]. Given the improved RA management over the past few decades, the aim of this study was to assess the prevalence of RA-specific lesions in cervical spine on radiographs and MRI and to correlate findings with clinical and laboratory parameters.

## 2. Materials and Methods

The study was approved by the Ethics Committee (no. KBT-3/2/2018). Conventional radiographs in three lateral views (maximum flexion, neutral (resting), and maximum extension) as well as MRI of the cervical spine of 240 consecutive patients with confirmed RA performed at our institution from 1 January 2010 to 28 February 2018 were retrospectively analyzed. 

Patients were qualified for imaging by clinicians based on symptoms suggesting cervical spine involvement (e.g., neck pain, limited motion of the cervical spine, numbness of upper extremity) and/or long disease duration. Patients with osteoarthritis served as a control group. Exclusion criteria included past surgery on cervical spine and traumatic lesions. The maximal time interval between radiography and MRI did not exceed 60 days.

MRI was performed in a 1.5 T Siemens Avanto with the use of an 8-channel neck coil. The MRI protocol included sagittal T1-weighted (w), T2-w, T2-w TIRM, axial T2-w, and coronal T2-w sequences as well as postcontrast axial and sagittal T1-w with fat saturation (fs) sequences in some patients. 

MRI and radiographic lesions were scored in a binary way: 0—absence of pathology, 1—presence of pathology. On radiographs, the following lesions were scored: (1) in the whole cervical spine: demineralization, cysts and erosions, bone ankylosis, and spinal stenosis; (2) at the C1/C2 level: dens erosions, anterior atlanto-axial subluxation (AAS), basilar settling (vertical AAS), posterior AAS; and (3) at the C2–C7 subaxial level: subluxations (SAS) (Table 1).

On MRI, bone marrow edema (BME) in the cervical spine, cysts, erosions, bone ankylosis, spinal stenosis, and the compression of brain stem or spinal cord and myelopathy were evaluated. In addition, at the C1/C2 level joint effusion, inflammatory pannus and lateral subluxation were reported, and at the C2–C7 level, the presence of SAS was reported (Table 1).

Anterior AAS was reported when the distance between the posterior surface of the anterior arch of the atlas and anterior margin of the dens exceeded 3 mm [1]. The anterior atlanto-dental interval was calculated in neutral position and if eligible in flexion. A posterior atlanto-dental interval (PADI) less than 14 mm required prompt neurosurgical consultation (potential cord compromise, compression) [1]. Lateral AAS was diagnosed when there was >2 mm displacement or an asymmetry of dens in relation to C1 body [6]. Basilar settling (i.e., vertical AAS, cranial settling) was considered when the apex of dens was located >4.5 mm above McGregor’s line [7]. SAS was considered when there was >2 mm displacement between adjacent vertebrae [8].

For assessment of cervical spinal stenosis on radiography, the canal to body ratio (Torg-Pavlov ratio) was used. The ratio is calculated on sagittal planes dividing the diameter of the spinal canal by the diameter of the vertebral body. A ratio of below <0.8 is considered cervical spinal stenosis [9]. Spinal stenosis on MRI was diagnosed when the AP diameter of the spinal canal was lower than 10 mm [10]. Myelopathy was reported when there was a high signal of spinal cord in T2-w turbo inversion recovery magnitude (TIRM) images.

Every radiograph and MRI scan was assessed in a blinded and randomized manner by two independent radiologists—ISS (20 year of experience) and MK (senior radiology resident). Both readers work in a reference center. The obtained data were analyzed for inter-reader reliability.

In each case, the following demographic, clinical, and laboratory data were collected: age, gender, disease duration, current medical treatment, serum concentration of C-reactive protein (CRP, mg/L), erythrocyte sedimentation rate (ESR, m/h), antinuclear antibodies (ANA) titer, seropositivity for anti-cyclic citrullinated peptides (anti-CCP antibodies), and RF. For ANA titer, higher than or equal to 1:160 was considered as significant. A concentration of >17 IU/mL was considered as positive for anti-CCP antibodies, while >34 IU/mL was considered positive for RF.

### Statistical Analysis

The statistical analysis was performed using SPSS software. The Shapiro–Wilk test was used to assess the distribution of continuous variables. Normally distributed data are expressed as mean ± standard deviation. Non-normally distributed data are presented as median and interquartile range (IQR). Student’s t-test and Mann–Whitney U-test were used to evaluate data when appropriate. Categorical Chi-squared test and Fisher’s exact test were used to assess nominate data when appropriate. *p*-values < 0.05 were considered as significant. The sensitivity and specificity of radiography for basilar settling and dens erosions were calculated relative to the gold standard of MRI and for MRI for anterior AAS and SAS relative to the gold standard of dynamic radiography. The inter-reader reliability kappa value was calculated using McHugh’s assumptions [11]. The level of agreement was classified as follows: almost perfect (kappa value above 0.90), strong (0.80–0.90), moderate (0.60–0.79), weak (0.40–0.59), minimal (0.21–0.39), none (0–0.20).

## 3. Results

Overall, 240 patients aged 23–86 years were enrolled (median age 62.0; IQR 53.0–69.0; 86% female), with a median duration of disease of 14.0 years; IQR 7.0–23.0. The mean age of RA onset was 42.7 ± 14.4 years.

Moreover, 198 patients with diagnosis of cervical spine osteoarthritis were recruited to serve as a control group. They were age and gender matched to the RA group (83% female, median age 62.0, IQR 54.0–70.0).

In the RA group, out of all 240 included patients, functional lateral radiographs were performed in 160 patients, while lateral neutral projection only was performed in 80 patients. Non-contrast MRI was performed in 168 patients and contrast-enhanced MRI was performed in the remaining 72 patients.

In the control group, functional radiographs were performed in 96 patients, and neutral projection only was performed in 102 patients. Twenty-one patients from the control group had post-contrast MRI of the cervical spine.

**Figure 1:**
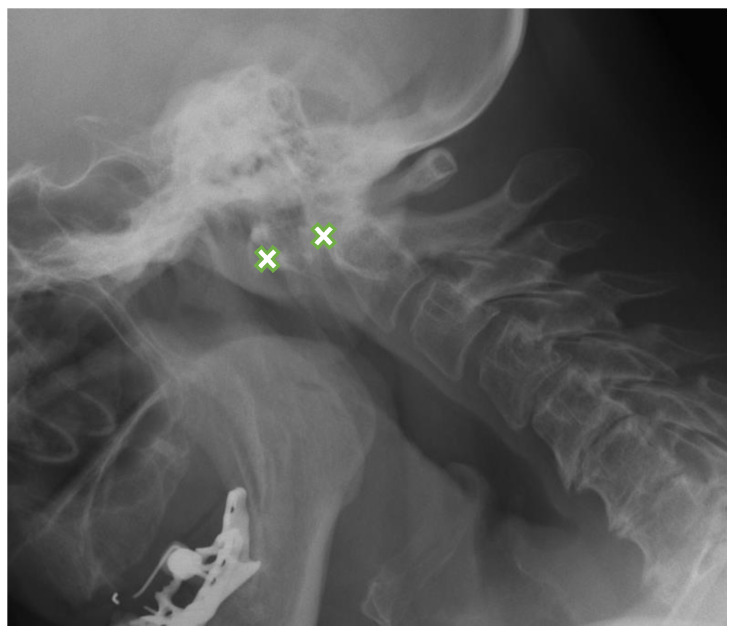
Lateral flexion view radiograph in a 63-year-old female with rheumatoid arthritis shows anterior AAS-8.8 mm (between crosses). AAS: atlanto-axial subluxation.

Out of 240 patients in the RA group, 179 (75%) had RA-related abnormalities of the cervical spine seen on radiographs and/or MRI. The most common lesions were anterior AAS (Figure 1) diagnosed in 140 patients (58%) with radiographs and in 78 (33%) with MRI, SAS seen in 139 patients (58%) on radiographs and in 102 patients (43%) with MRI, and demineralization diagnosed in 114 patients (48%) entirely with radiographs (Table 1).

**Figure 2:**
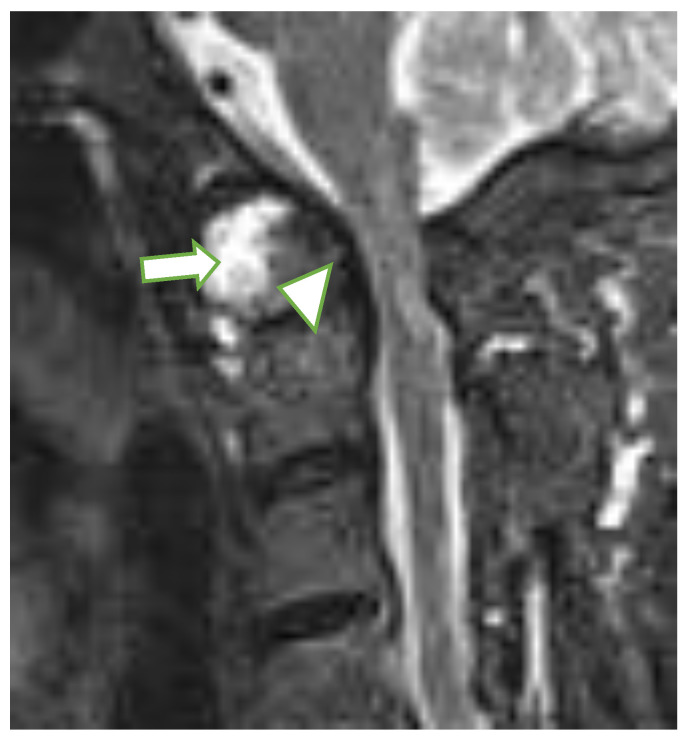
Sagittal MRI, T2-w TIRM image in a 65-year-old female with rheumatoid arthritis shows dens erosions (arrowhead) and periodontal effusion and pannus (long arrow). MRI: magnetic resonance imaging, TIRM: turbo inversion recovery magnitude.

**Figure 3:**
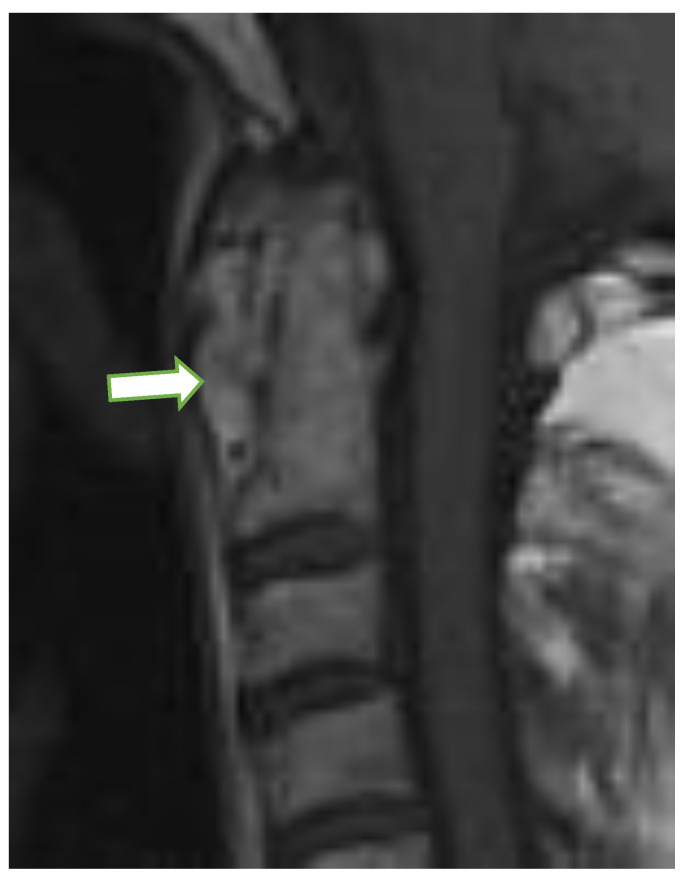
Sagittal postcontrast MRI, T1-w image in a 76-year-old female with rheumatoid arthritis with pannus formation in periodontal area (arrow). MRI: magnetic resonance imaging.

At the C1/C2 level, the most frequently diagnosed abnormalities (apart from anterior AAS) were (Table 1) vertical AAS (25 [10%] seen on MRI, 27 [11%] on radiography), posterior AAS (11 [5%]) and lateral AAS (7 [3%]); the latter two were observed only with MRI. Dens erosions were seen in 36 patients (15%; Figure 2) on MRI, while radiography showed dens erosions in 11 subjects. With MRI only, pannus was diagnosed in 50 patients (21%; Figure 3), periodontal effusion in 26 (11%), BME in 11 (5%), and contrast enhancement of bone (osteitis) and/or synovium in 12 (5%) patients. MRI showed brain steam compression in eight patients (3%).

Among 140 cases of anterior AAS, 43 were seen on neutral lateral view and confirmed by functional radiography, while 69 of them were missed on neutral projections and diagnosed using additional views. Overall, 62% patients of anterior AAS were visible only on functional radiographs.

At the subaxial level, the most common lesion was SAS (139 (58%) seen on radiography, 102 (43%) on MRI).

The radiography sensitivity compared to MRI in detection of the basilar settling was 76%, while specificity was 96%, respectively. The sensitivity of radiography in the detection of dens erosions is 25%, while specificity was 99%. The sensitivity of functional radiography compared to static MRI in the diagnosis of anterior AAS was 50.7%, while specificity is 93%. In the case of SAS, the sensitivity of MRI was 54% and specificity was 73%.

At least one method discovered ankylosis in 23 patients (10%) (MRI in 14 patients, radiography in 19 patients). Spinal stenosis was seen with MRI in 93 patients (39%) and with radiography in 63 patients (26%). MRI showed cervical spine compression in 72 patients (30%) and cervical myelopathy in 14 patients (6%).

MRI revealed BME in 11 patients (5%), effusion in one patient, pannus formation in one patient, and contrast enhancement of bone (osteitis) and/or synovium in two patients. Spinous process erosions were seen in 14 patients (6%) and demineralization was seen in 114 (48%).

Most (90%) patients were taking at least one DMARD, two or more drugs were taken by 105 patients (71%), and 20 patients were on biological treatment.

Anti-CCP antibodies were present in 78 patients (78%), RF was present in 51 (51%), and ANA was present in 34 (34%). The median CRP value was 15 mg/L (IQR 7.0–30.3), while the median ESR value was 33 mm/h (IQR 16.3–56.8). Table 2 presents detailed clinical and laboratory data.

Cervical spine involvement was linked with longer duration of RA (15 years (range: 9.0–27.0) vs. 9 (4.0–16.0, *p* = 0.007), presence of RF (39 (60%) vs. 12 (35%); *p* = 0.010), elevated CRP (19.0 (7.0–32.0) vs. 11.5 (6.3–19.8); *p* = 0.016) and ESR (35.0 (19.0–58.0) vs. 28.0 (13.3–48.8); *p* = 0.025). The longer duration of RA was associated with the presence of subaxial subluxation (30 years (15.0–40.0) vs. 13 (6.0–21.0); *p* = 0.005), anterior AAS (15 years (10.0–27.0) vs. 9.5 (3.0–18.0); *p* = 0.005) and basilar settling (17 years (9.0–27.0) vs. 10 (5.0–16.0); *p* = 0.003). Basilar settling prevalence was lower in patients treated with methotrexate (6% vs. 17%; *p* = 0.041). Vertical AAS was also linked with the presence of demineralization (18% vs. 4%, *p* = 0.001). No other significant associations were found between detected abnormalities and treatment.

Comparing RA and the control group, the majority of lesions had statistically higher prevalence in RA (Table 1). For the spinal stenosis, myelopathy, cervical cord and brain stem compression, as well as subaxial lesions (BME, effusion, pannus, and contrast enhancement of bone (osteitis) and/or synovium), no statistically significant differences were found between the RA group and the control group.

The overall inter-reader reliability (for the reference method) was usually almost perfect (kappa value >0.90) or strong (0.80–0.90). For anterior AAS (0.79), demineralization (0.76), and subaxial lesions (effusion—0.66; pannus—0.66, and contrast enhancement—0.67), the agreement was moderate (Table 1).

## 4. Discussion

The current study confirmed the high prevalence of cervical spine pathologies in RA patients. Out of 240 included patients, 75% developed cervical spine abnormalities, with 71% on DMARDS therapy.

The most common lesions were anterior AAS (58%), followed by SAS (58%) and bone loss (48%). The cervical spine involvement was linked with long-standing RA, raised inflammatory markers, and RF seropositivity. Vertical AAS, anterior AAS, and subaxial subluxation developed late in the course of RA, while the remaining lesions including dens erosions may occur anytime.

The most serious complication of cervical spine arthritis is subluxations. Recent studies showed that anterior AAS was present in 18–32% of patients [8,12,13], whereas in our study, it was diagnosed in 58% of patients by radiographs, most likely because the study was conducted in a reference center for rheumatic diseases. Usually, AADI (anterior atlanto-dental interval) higher than 6 mm is considered for surgery [14]. In the current study, inter-reader agreement for anterior AAS was moderate (kappa—0.79). This usually resulted from suboptimal visualization/superimposition of anatomical structures due to destructive lesions of dens or border cases where the AADI was approximately 3 mm.

The second most commonly diagnosed abnormality was SAS, and it was again more common in our study (139 patients [58%]) than in those published by other authors (6–16%) [8,12]. SAS is caused by destructive changes in intervertebral, uncovertebral, and apophyseal joints as well as ligamental damage. In the current study, SAS developed in patients with a median of 30 years of history of RA. As other subluxations, SAS may also lead to myelopathy. The severity of subaxial myelopathy may be associated with vertebral or intervertebral disc destruction, the spinous process, or apophyseal joint erosions. Long-standing RA, younger age, and treatment with corticosteroids are risk factors for developing subaxial myelopathy [15].

Vertical AAS occurred in 10% of the RA patients, which is a similar prevalence to that observed in previous studies [8,12]. The assessment of vertical AAS is challenging on classic radiography due to the superimposition of anatomical structures and bone damage in the course of RA. Several methods are used to assess vertical AAS: Chamberlain, Clark, McRae, McGregor, Redlund-Johnell, Ranawat, Fischgold-Metzger, Wackenheim, and Kauppie-Sakaguchi [7,16,17,18,19,20,21,22,23]. In the current study, the McGregor method was used, and vertical AAS was considered when the apex of dens was located >4.5 mm above McGregor’s line [7]. Riew et al. revealed that no method is ideal [24]. The authors suggest that combined measurements with Clark, Ranawat, and Redlund-Johnell methods are preferable. If any of these three methods is positive for the detection of vertical AAS, the sensitivity reaches 94% [24].

Care must be taken in patients with vertical subluxation, since in significant ligamental damage with the coexistence of anterior and vertical AAS, the anterior distance between the C1 arch and dens (AADI) may decrease and even normalize, causing pseudostabilization.

Other types of AAS, such as lateral or posterior, are rare. Only 5% of included patients developed lateral AAS, while 3% developed severe posterior AAS. The prevalence of these types of AAS is similar to previous studies [6]. Posterior AAS is usually caused by odontoid erosions or fracture, while lateral AAS is caused by rotatory deformations. Posterior AAS is an indicator for surgery. PADI less than 14 mm requires prompt neurosurgical consultation due to high risk of spinal cord compressions and is a poor prognostic factor leading to neurological deficit [14,25].

Dens and spinous process erosions are a hallmark of RA. Dens erosions were seen in 15% of our patients, in line with published data by Olah et al. [12], which reported a prevalence of 16%. The prevalence of spinous process erosions in our study was 6%. Moreover, correlation between dens erosions and destruction in peripheral joints was found [12].

In the current study, at the C1/C2 level, pannus was seen in 21% of patients, showing postcontrast enhancement in 5% of the patients, periodontal effusion in 11%, and BME in 5%. Another study showed similar results; the prevalence of pannus formation was almost 25% [13]. Carotti at al. [26] found cervical spine arthritis in 24% of patients in early RA (less than a year from the diagnosis). The vast majority of lesions included BME, pannus, and dens erosions [26]. However, in our study, no link between BME, pannus formation or effusion, and duration of disease was confirmed.

Demineralization was found on radiographs in almost half of the patients. Han et al. suggested that patients with low bone mineral density (BMD) and lower body mass index (BMI) have a higher risk of AAS [27]. Rossini et al. suggested that osteoporosis may be an independent risk factor for bone erosions in RA [28]. In the current study, bone demineralization was positively linked to vertical AAS only (*p* = 0.001). Although the major adverse effect of steroid treatment is reduced BMD, the recent metanalysis revealed that patients with early RA after 24 months of corticosteroid therapy have no changes in BMD [29]. For demineralization, the interobserver kappa value was moderate (0.76). However, radiography is the only axillary method, while the gold standard for assessment of BMD is dual-energy X-ray absorptiometry.

Apophyseal bony ankylosis in not a specific feature of RA, but it can be observed with long duration of disease. In the current study, cervical spine ankylosis was diagnosed in 10% of the patients. Iizuka et al. [30] found cervical ankylosis in 24% of RA patients, and it most commonly affected the atlanto-occipital joint. At the subaxial level, ankylosis may be the risk factor for instability and stenosis and may even lead to myelopathy. At the upper cervical level, patients with ankylosis had neurological impairment [30].

Lesions of spinal cord and brainstem were assessed on MRI and included spinal stenosis (39%), cervical cord compression (30%), cervical myelopathy (6%), and brainstem compression (3%). It is speculated that the incidence of spinal compression is underestimated in clinical practice, which is mainly due to nonspecific symptoms or a lack of symptoms, and therefore, it may lead to a sudden death [31]. If not operated, myelopathy deteriorates in 76% of patients. Within 3 years, all patients become bedridden, and the cumulative survival rate after 7 years was 0% [32]. When operated, 71% of patients with paralysis improved neurologically [25]. Neva et al. reported that 2% of patients with RA died due to cervical spine compression, resulting in sudden death, postoperative complications, and paraparesis or quadriparesis [2]. Cervical myelopathy is usually caused by mechanical compression or vascular ischemia [33]. With MRI, it manifests as a focal area of increased signal in fluid-sensitive images; however, very early changes might remain undetected. Recently, apparent diffusion coefficient (ADC) allows the early identification of cervical cord pathologies in patients with anterior AAS and normal MRI. This parameter may be useful for spinal surgery qualification [34]. Compared to a control group of patients with osteoarthritis, there were no statistically significant differences in prevalence of spinal stenosis, cervical cord compression, cervical myelopathy, and brain stem compression between patients with RA and osteoarthritis. In RA, these lesions are caused mainly by subluxations, while in osteoarthritis, they are caused by degenerative changes such as osteophytes or disc protrusion/extrusion.

The current study confirmed that a long duration of RA, elevated CRP concentration, and elevated ESR level as well as the presence of RF are associated with cervical spine arthritis. Other studies also showed that female sex, long treatment with steroids, extensive peripheral joint involvement, and the presence of anti-CCP antibodies may be risk factors for cervical spine arthritis [4,12]. Although raised CRP and ESR are seen in RA and correspond with disease activity, both markers are nonspecific and may be elevated in other inflammatory conditions or RA complications. Furthermore, a recent study found histological features of inflammation in the synovium of the knee in RA patients with normal CRP. Thus, relying exclusively on inflammatory markers for disease severity score may not be ideal [35].

The first-line treatment for RA is methotrexate. One-third of patients on methotrexate did not show radiological progression, and this positive effect is even greater in combination with other drugs [36]. The current study showed a lower prevalence of vertical AAS in patients treated with methotrexate. The intensive use of a combination of three DMARDs (methotrexate, sulfasalazine, hydroxychloroquine) with prednisolone limited the development of anterior AAS [37]. Regarding biological treatment, Salii et al. reported that infliximab therapy reduced periodontal pannus area and spinal cord edema [38]. Sandstrom et al. [39] focused on the prevalence of cervical spine involvement after 10 years of follow-up during triple DMARD therapy with prednisolone in patients who also received infliximab as part of a double-blind randomized study. The incidence of cervical spine involvement was as low as 4.7% regardless of infliximab use [39]. Biological agents prevented the development of new cervical spine lesions but did not inhibit the progression of preexisting involvement. Thus, the progressive nature of RA may still be uncontrollable [5]. Other authors reported lower prevalence of cervical spine involvement in the era of biological treatment, but their study failed to determine the role of biological agents [40]. In addition, metaloproteinase-3 levels predicted bone damage in treated patients [41,42]. Therefore, patients diagnosed with cervical spine instability tend to have progressive disease even under appropriate medical treatment, whereas patients without cervical involvement may benefit from the treatment [40,41,42].

Radiography and MRI are complementary methods. Radiographs remain a first-line method in imaging of the cervical spine. Neutral view in a standing position, supplemented by dynamic projections are superior to standard MRI (without flexion and extension) in the diagnosis of anterior and posterior AAS and SAS. However, MRI with no superimposition of anatomical structures allows better visualization of vertical AAS and dens erosions. The current study showed that functional projections detected 62% of all anterior AAS, which were not seen in the neutral position. This is similar to previous observations [43]. The sensitivity of dynamic radiography compared to static MRI in the supine position in diagnosis of anterior AAS was 51% and specificity was 93%. For SAS, the sensitivity of MRI was 54% and specificity was 73% compared to radiography. In the case of anterior AAS, Hung et al. list a number of features that are seen on MRI and may suggest anterior AAS, namely: dens erosions, anterior atlantoaxial joint titling (situation where the anterior tubercle of C1 and the anterior cortex of the odontoid process are not parallel), effusion, lateral facet arthropathy, abnormalities of spinolaminar line, and cervical myelopathy [44]. Otherwise, MRI is used to assess the activity of inflammation and complications, including other directions of subluxation (lateral, vertical, rotatory, mixed) and is the method of choice to visualize spinal cord and brainstem lesions such as compression or myelopathy [45]. Our study showed the superiority of MRI in the diagnosis of dens erosions and vertical AAS. Radiography showed 25% sensitivity and 99% specificity as a diagnostic modality for dens erosions and 76% sensitivity and 96% specificity for the detection of vertical AAS. The major limitation of radiography is the superimposition of anatomical structures used as reference points for measurements, which obscures anatomical landmarks.

The major advantage of the current study was the large, single-center cohort of 240 patients diagnosed with RA. In the study, both MRI and radiographic methods were analyzed, and the pros and cons of each modality were shown. The main limitation was the retrospective nature of the analysis.

## 5. Conclusions

The current study showed that RA-related lesions occur in most RA patients (75%), and they most frequently include anterior AAS (58%) and SAS (58%) followed by bone loss (48%). Dynamic radiographs and MRI are complementary and should both be performed for optimal diagnosis. Long disease duration, elevated inflammatory markers, and RF seropositivity were positively linked with abnormalities. In this cohort, the prevalence of vertical AAS was reduced in patients taking methotrexate, whereas no effect on remaining subluxations or RA features was noted.

## Figures and Tables

**Table 1:** Prevalence of pathologies in the study group. * MRI results are presented as a gold standard, # Radiography findings are presented as a gold standard as a study providing dynamic assessment of subluxations, contrary to static MRI, $ Ankylosis is present, when detected by at least one method. AAS: atlanto-axial subluxation, BME: bone marrow edema, MRI: magnetic resonance imaging, ns: not significant (difference between rheumatoid arthritis and control group), SAS: subaxial subluxation.

	MRI Lesions	Radiographic Lesions	Percentage	Control n (%)	Interobserver Kappa Value
C1/C2 level					
BME	11	not applicable	11 (5%)	1 (0.5%)	0.91
Effusions	26	not applicable	26 (11%)	5 (2.5%)	0.89
Pannus	50	not applicable	50 (21%)	1 (0.5%)	0.93
Contrast enhancement	12	not applicable	12 (5%)	0 (0%)	0.83
Dens erosions	36	11	36 (15%) *	2 (1%)	0.89
Anterior AAS	78	140	140 (58%) #	7 (3.5%)	0.79
Posterior AAS	7	0	7 (3%)	0 (0%)	0.83
Lateral AAS	11	not applicable	11 (5%)	0 (0%)	0.84
Vertical AAS	25	27	25 (10%) *	2 (1%)	0.93
Brain steam compression	8	not applicable	8 (3%)	2 (1%) ns	0.95
Subaxial C2-C7 level					
BME	11	not applicable	11 (5%)	6 (3%) ns	0.86
Effusions	1	not applicable	1 (0.4%)	0 (0%) ns	0.67
Pannus	1	not applicable	1 (0.4%)	0 (0%) ns	0.67
Contrast enhancement	2	not applicable	2 (1%)	2 (1%) ns	0.66
SAS	102	139	139 (58%) #	78 (39%)	0.83
Whole cervical spine C1-C7					
Ankylosis	14	19	23 (10%) $	3 (1.5%)	0.87
Spinous process erosions	0	14	14 (6%)	0 (0%)	0.83
Demineralization	not applicable	114	114 (48%)	35 (18%)	0.76
Myelopathy	14	not applicable	14 (6%)	7 (3.5%) ns	0.92
Cervical spine compression	72	not applicable	72 (30%)	59 (30%) ns	0.95
Spinal stenosis	93	64	93 (39%) *	72 (36%) ns	0.92
Total	138 (58%)	159 (66%)	179 (75%)	95 (48%)	

**Table 2:** Characteristics of RA group. ANA: antinuclear antibodies, CCP: cyclic citrullinated peptides, CRP: C-reactive protein, ERP: erythrocyte sedimentation rate, MRI: magnetic resonance imaging, RA: rheumatoid arthritis, RF: rheumatoid factor.

Rheumatoid Arthritis	Cervical Spine Lesions on Radiographs and/or MRI (*n* = 179)	No Abnormalities on Imaging (*n* = 61)	*p*
Age (years)	61.0 [52.0–68.0]	59.5 [50.5–66.8]	0.491
Sex (female; %)	154 (86%)	53 (87%)	0.868
Age at onset (years)	41.8 ± 13.6	45.3 ± 16.3	0.229
Disease duration (years)	15.0 [9.0–27.0]	9.0 [4.0–15.8]	0.007
CRP (mg/mL)	19.0 [7.0–32.0]	11.5 [6.3–19.8]	0.016
ESR (mm/h)	35.0 [19.0–58.0]	28.0 [13.3–48.8]	0.025
ANA positivity *n* (%)	20 (31%)	14 (39%)	0.408
RF positivity *n* (%)	39 (60%)	12 (35%)	0.010
Anti-CCP positivity *n* (%)	54 (83%)	24 (67%)	0.060

## Data Availability

The study data may be available on request.
